# Optimization and qualification of an Fc Array assay for assessments of antibodies against HIV-1/SIV

**DOI:** 10.1016/j.jim.2018.01.013

**Published:** 2018-04

**Authors:** Eric P. Brown, Joshua A. Weiner, Shu Lin, Harini Natarajan, Erica Normandin, Dan H. Barouch, Galit Alter, Marcella Sarzotti-Kelsoe, Margaret E. Ackerman

**Affiliations:** a Thayer School of Engineering, Dartmouth College, Hanover, NH 03755, United States; b Ragon Institute of MGH, MIT, Harvard University, 149 13th St, Charlestown, MA 02129, United States; c Beth Israel Deaconess Medical Center, Boston, MA 02215, United States; d Duke University Medical Center, 2812 Erwin Rd., Suite 301, Erwin Terrace II, Durham, NC 27705, United States

## Abstract

The Fc Array is a multiplexed assay that assesses the Fc domain characteristics of antigen-specific antibodies with the potential to evaluate up to 500 antigen specificities simultaneously. Antigen-specific antibodies are captured on antigen-conjugated beads and their functional capacity is probed via an array of Fc-binding proteins including antibody subclassing reagents, Fcγ receptors, complement proteins, and lectins. Here we present the results of the optimization and formal qualification of the Fc Array, performed in compliance with Good Clinical Laboratory Practice (GCLP) guidelines. Assay conditions were optimized for performance and reproducibility, and the final version of the assay was then evaluated for specificity, accuracy, precision, limits of detection and quantitation, linearity, range and robustness.

## Highlights

• Standardized array-based characterization platform for polyclonal antibodies
• Qualification of a biophysical proxy for antibody effector function
• Antibody recognition of antigen and by Fc receptors is captured.
• Enables systematic serologic studies of NHP and human antibody samples.

## 1 Introduction

The Fc Array assay is a platform by which the effector capacity of antibody samples is assessed via evaluation of antigen recognition and Fc receptor ligation in a highly multiplexed manner (Brown et al., 2017). Given the importance of antibody effector functions in vivo, and the broad range of phenotypic variability present in antibodies from different subjects, this method was developed to broadly profile these differences, and to provide a biophysical assay alternative to traditional cell-based effector function assays, such as those developed for antibody dependent cellular cytotoxicity (ADCC), complement-dependent cytotoxicity (CDC), and antibody dependent cellular phagocytosis (ADCP).

The purpose of assay qualification is to ensure reproducibility of data both within and between studies, and to facilitate acceptance of data by external partners and regulatory agencies. In particular, since the Fc Array assay (Brown et al., 2017) is designed to potentially contribute to clinical trial endpoints, it is important that assay parameters are designed to facilitate compliance with Good Clinical Laboratory Practice (GCLP) (Stevens, 2003; Sarzotti-Kelsoe et al., 2009; Ezzelle et al., 2008). GCLP was initially designed by the British Association of Research Quality Assurance (BARQA) in 2003 and was later expanded upon by the NIH/NIAID/DAIDS in 2008, and harmonized in 2009 to provide a regulatory framework for laboratories performing endpoint assays for HIV-1 human clinical trials (Stevens, 2003; Sarzotti-Kelsoe et al., 2009; Ezzelle et al., 2008). The process of converting laboratories into GCLP-compliant entities includes initial laboratory assessments and GCLP training, establishment of standard operating procedures (SOPs), quality management systems and study plans, quality control of equipment and reagents, optimization and validation of applicable assays, and regular laboratory audits and corrective action programs (Todd et al., 2014).

The goal of optimization is to determine the conditions necessary to make the assay as reliable and effective as possible. Relevant experimental data, as well as the best judgment of the scientists involved can then be used to establish standard operating procedures (SOPs) and define the acceptance criteria during assay qualification. Assay qualification can provide documented evidence that the method is operating accurately and consistently, is sensitive enough for its intended application, and is suitable for its intended purpose, i.e. the method is “fit for purpose”.

Qualifying an assay consists of evaluating the applicability of the parameters described in the Harmonized Tripartite Guideline to Validation Of Analytical Procedures: Text And Methodology (Validation of analytical procedures: text and methodology Q2(R1), 2005) guidelines for relevance to the assay and its intended use, and defining acceptable ranges for parameters such as accuracy, precision, limit of detection, limit of quantitation, specificity, linearity and range, robustness, and system suitability. Qualification parameters were determined for the Fc Array with reference to both human and non-human primate (NHP) reagents. This assay has recently been provided as a service for evaluation of human and non-human primate (NHP) HIV/SIV vaccine studies (Barouch et al., 2015; Vaccari et al., 2016; Bradley et al., 2017), and therefore its standardization and qualification are reported to best support these efforts. Here we describe the optimization and qualification of the Fc Array assay as it is performed at the Dartmouth Antibody Laboratory (dAbl) site at Dartmouth College. In addition to in-house experiments, dAbl participates in a Luminex proficiency testing program via the External Quality Assurance Program Oversight Laboratory (EQAPOL) at Duke University (Lynch et al., 2014). While the multiplexed cytokine quantitation assay used is not strictly analogous to the Fc Array assay, since it differs in bead, analyte, and detection reagent composition, it nonetheless offers a valuable external check on instruments and personnel in their ability to perform an assay utilizing similar principles and the same equipment to pass preset conditions.

## 2 Materials and methods

Given prior publication of the assay method (Brown et al., 2017), the focus of this manuscript is to give an overview of the formal optimization and standardization of this assay, rather than an exhaustive listing of all optimization experiments or a detailed analysis of the method itself.

### 2.1 Preparation of antigen-coupled array microspheres

HIV antigens were coupled to magnetic carboxylated fluorescent beads (Luminex Corporation) as described previously (Brown et al., 2017). Briefly, a total of 5 million carboxylated beads (400 μl) were covalently coupled to 25 μg of antigen using a two-step carbodiimide reaction, and then blocked and suspended in PBS (Phosphate Buffered Saline) -TBN (PBS-1×, 0.1% BSA, 0.02% Tween 20, 0.05% Sodium Azide, pH 7.4, Teknova). The coupled beads were counted (TC-10 cell counter, BioRad) and stored at −80 °C for up to 6 months or at 4 °C for up to 1 month prior to use. Antigen purity was known for most but not all materials used, and was generally >90% as assessed by HPLC, SDS-PAGE, or Edman sequencing/amino acid analysis/mass spectroscopy. Consistent performance of differing antigen or conjugation batches is ensured by meeting acceptance criteria in bridging experiments.

### 2.2 Preparation of clinical plasma/serum antibody samples

Human subjects were recruited from Ragon Institute and Global Solutions for Infectious Disease cohorts and included placebo and vaccine recipients, healthy, acute, and chronically HIV infected subjects, as well as controllers, individuals able to maintain long-term suppression of virus in the absence of anti-retroviral therapy. The study was approved by the Massachusetts General Hospital and Dartmouth College Institutional Review Boards, and each subject gave written informed consent. Rhesus plasma samples were obtained from immunization studies approved by the appropriate Institutional Animal Care and Use Committees. Individual plasma samples were barcoded and aliquoted into 384-well master plates, with the codes read into custom database software to generate plate layouts. Because of the sensitivity of this assay, multiple serial dilution plates (e.g., 10×, 100×, 1000× in PBS) were often made with the same layout to achieve the final assay concentration, and were frozen for up to 6 months prior to use. Studies with the same samples were generally set up from the same master plate for multiple detection reagents to minimize the possibility of operator error. These master plates were stored at 4 °C for up to six weeks without significant effect on assay results.

### 2.3 Preparation of Fc receptors (FcR)

Human FcγRs (FcγRI, FcγRIIa, FcγRIIb, FcγRIIIa, FcγRIIIb) were produced via transient transfection in human embryonic kidney (HEK) 293F cells grown in Freestyle media (Invitrogen) using 25 kD branched PEI (PolySciences), and purified via immobilized metal affinity chromatography (IMAC) followed by size exclusion chromatography (SEC) as described previously (Boesch et al., 2014). Size and purity of all recombinant protein was confirmed by SDS-PAGE, and consistent performance was ensured via meeting acceptance criteria in bridging experiments.

Human and Rhesus FcγRs were reformatted with a C-terminal GGG-AVI-His tag in order to facilitate site-specific biotinylation. Expression and purification were carried out as described for the non-AVI variants. Biotinylation was carried out as described previously (Brown et al., 2017). Concentration of biotinylated FcγRs was determined by absorption at 280 nm, and proteins were stored at −80 °C for up to twelve months. Non AVI-tagged FcRs such as C1q and MBL were biotinylated using EZ-Link Sulfo-NHS-SS-Biotin (Pierce) at a molar ratio of 5 mols biotin per mol of protein and used as streptavidin tetramers as described previously (Brown et al., 2017).

### 2.4 External proficiency testing (EQAPOL)

EQAPOL testing evaluates performance twice yearly on a standardized commercial multiplex assay kit with standards to quantify human IFNγ, TNFα, IL-6, IL-10 and IL-2, and a series of recombinant cytokine-spiked human serum samples provided by EQAPOL, as described previously (Lynch et al., 2014).

### 2.5 Fc Array protocol

The Fc Array was carried out as described previously (Brown et al., 2017). Briefly, 40–45 μl of bead mastermix (containing a total of 500 beads per type per well) was added to 5–10 μl of diluted antibody sample and incubated for 2 h (±12 min) at room temperature with orbital shaking (1000 rpm, IKA 3208001 MTS 2/4). After primary incubation the plates were washed 6× on a plate washing system (BioTek 405), and the beads resuspended in 50 μl of detection reagent per well. Following a 1 h (±6 min) incubation with XYZ shaking the plates were washed 6× in Luminex sheath fluid and the beads resuspended in 40–50 μl sheath fluid per well. Robotic (EpMotion 5075, Eppendorf) or manual (multichannel pipettes, Rainin) methods were shown to be comparable and used interchangeably for plate setup and addition of detection reagent and sheath fluid. A Luminex array reader (FlexMap 3D, Luminex Xponent 4.2, Luminex Corp.) detected the microspheres and level of PE fluorescence was measured to calculate a Median Fluorescence Intensity (MFI). HIVIG (3957, AIDS Reagent Program) was a positive control and utilized as a means to track plate variation, and HuIgG (I2511, Sigma) served as an HIV-1 negative control. An in-house SIVIG standard consisting of pooled serum from several moderate titer rhesus macaque samples served as an SIV positive control.

### 2.6 Data analysis

Raw data were analyzed in the Bio-Plex Manager 5.0 software, or a proprietary database constructed for dAbl, for assignments of standard, controls, samples, etc. Data were exported as Excel or csv files and input into PRISM or the R statistical environment for analysis. To fit dose response curves, the MFI and antibody concentration were log transformed and fit with a non-linear regression using a variable slope four-parameter function. Correlation coefficients (R^2^) and best fit line slopes were calculated using linear regression in PRISM. Limit of detection (LOD) values were determined as lowest tested quantity of positive control for which MFI signal was greater than the average + 3 standard deviation of technical negative measurements and limit of quantitation (LOQ) values as average + 10 standard deviations of negatives.

## 3 Results

### 3.1 Identification of critical parameters

Critical parameters for an assay are those for which even relatively small variations can cause a significant difference in assay results, and which are therefore important to monitor and control. In the case of the Fc Array, potential critical parameters include length of incubation periods, bead counts within individual wells, antibody sample types and dilutions, and production and storage conditions of antigens, antigen-conjugated beads, and custom FcγR detection reagents. Incubation periods have been limited to make the assay sufficiently high throughput and able to be performed by a single technician within a standard 8 h workday. Because of this constraint, for some conditions incubation lengths may be of insufficient duration to achieve equilibration of either antibody to the antigen beads in the primary incubation, or of detection reagents to bead-bound antibody in the secondary detection step. Thus, timed steps were carefully monitored in SOPs through the use of checklists, and acceptable variation criteria that have been shown not to affect the performance of the assay have been defined. Generally, variation of up to ±10% of the total incubation length was shown to be acceptable (data not shown). Other critical parameters were more amenable to optimization and will be discussed in more detail below.

### 3.2 Optimization of bead storage conditions

**Fig. 1:**
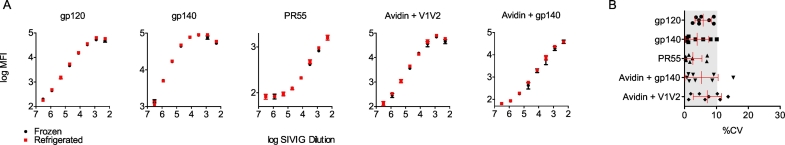
Bead freeze/thaw stability. A. Output MFI of an SIVIG dilution curve for frozen (black) and refrigerated (red) antigen bead sets across a panel of five representative antigen types including directly conjugated and avidin-captured antigen. Each condition was run in triplicate, and error bars represent the mean and standard deviation. B. Percent Coefficient of Variation (%CV) between refrigerated and frozen beads over the SIVIG dilution curve. Error bars represent the mean and standard deviation. Gray region denotes %CV ≤ 10%. (For interpretation of the references to colour in this figure legend, the reader is referred to the web version of this article.)

In prior iterations of this assay (Brown et al., 2012), antigen-conjugated beads were stored at 4 °C for up to two months prior to assay use, per the manufacturer's instructions for storage of the uncoupled beads. This condition was acceptable for relatively well-behaved proteins such as the commercial gp120s initially tested, but tended to give significant variation in signal (alternatively increasing or decreasing signal for different antigens) with less stable proteins. This variability was particularly pronounced for biotinylated antigens, for which capture with avidin-coated beads generally demonstrated the best performance. To reduce this variability, we tested the performance of both directly conjugated beads and those conjugated to avidin and used to capture biotinylated antigen through a freeze-thaw cycle. Microspheres were prepared following the bead conjugation SOP, then separated into frozen (−80 °C) and refrigerated (4 °C) batches. After 24 h, the frozen beads were then thawed and both sets of beads assayed against a triplicate SIVIG standard curve to determine the impact of the freeze-thaw process on bead/antigen quality (Fig. 1). The percent Coefficient of Variation (%CV) between bead handing conditions was generally below 10% for most measurements (Fig. 1B), and did not exceed that observed between technical replicates within handling conditions (data not shown). Due to the comparable quality of assay results using freeze-thawed beads, the protocol was updated such that conjugated beads are aliquoted and frozen at −80 °C immediately after coupling, and aliquots thawed as needed to perform the assay. Use of conjugated beads beyond a single freeze-thaw cycle was not tested and thus is not recommended.

### 3.3 Optimization of FcγR biotinylation

**Fig. 2:**
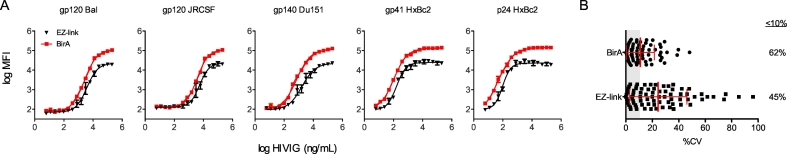
FcγR biotinylation optimization. A. Data points and extrapolated curve fits for HIVIG standard curves in which the FcγRIIIa F158 detection reagent was either biotinylated at primary amines (EZ-link, black) or site specifically at the C-terminus (BirA, red) across a panel of five representative antigen types. Each condition was assessed in triplicate and error bars represent the mean and standard deviation. B. Percent Coefficient of Variation (%CV) between receptor biotinylated using BirA or chemically at primary amines. The percent of measurements with a CV <10% (gray region) is listed at right. Error bars represent the mean and standard deviation. (For interpretation of the references to colour in this figure legend, the reader is referred to the web version of this article.)

Non-antibody detection reagents (such as FcγRs) undergo biotinylation and tetramerization via incubation with avidin prior to use in the Fc Array assay, consistent with the requirement of avidity for biological activity, and experimentally required due to their low monovalent affinities. Human FcγRs were initially produced with a C-terminal 6xHis-tag for purification and biotinylated on primary amines using a 5:1 M ratio of EZ-Link Sulfo-NHS-SS-Biotin. Previous optimization experiments determined that this ratio gave optimal signal; higher molar ratios of biotin eventually gave lower and even totally ablated signal, presumably due to biotinylation of lysine residues close to the Fc binding site (data not shown). These reagents worked acceptably well, but tended to exhibit undesirable levels of variability between individual biotinylation reactions and narrower dynamic ranges. In order to improve upon this potentially confounding factor, the human FcγRs were reformatted with a C-terminal GGG-AVI-6xHis tag to allow for site-specific biotinylation using the BirA enzyme (Chapman-Smith and Cronan Jr., 1999) while retaining the affinity tag for purification. In order to test the effectiveness of the AVI-tagged material, FcγR IIIA-R158 was biotinylated either site-specifically using BirA, or on primary amines with a 5:1 M ratio of EZ-Link Sulfo-NHS-SS-Biotin. These biotinylated FcγRs were then used as detection reagents with a 4:1 ratio of streptavidin-PE over a range of HIVIG concentrations (Fig. 2). Across all twelve antigens tested, the AVI-tagged detection reagents gave both a higher maximum signal and an extended linear range. Additionally, the site-specific biotinylation protocol resulted in a moderate reduction in the amount of variation seen between technical replicates. Rhesus macaque FcγRs were produced from the start using the AVI-tag system with site-specific biotinylation and have shown roughly comparable signal and range (within 2-fold) to the human receptors.

### 3.4 Optimization of number of bead counts per well

**Fig. 3:**
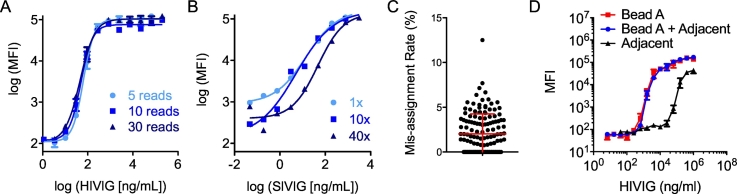
Effects of bead count. A. Representative HIVIG standard curve for one of a set of 13 antigen types when 5, 10, or 30 beads were read per well. Each condition was assessed in triplicate and error bars represent the mean and standard error. B. Representative SIVIG standard curve when 1, 10, or 40 times the standard number of beads were used for a single antigen type. C. Percentage of beads assigned to an adjacent but incorrect bead region per well (N = 144 wells). Error bar represents the mean and standard deviation. D. Standard curves for individual and combinations of spectrally adjacent bead sets. Each condition was assessed in triplicate and error bars represent the mean and standard error.

In order to reduce assay cost and competition for scarce antibody specificities that may cross-react to multiple antigens used, previous rounds of optimization had identified 500 beads per type per well as the optimal starting bead concentration with a minimum of 30 bead reads per type per well collected. Using these conditions, low bead counts (<30) are occasionally observed, but may still provide valuable data. To establish a minimum bead count for reliable results, duplicate HIVIG standard curves were assayed on a gp41 HXBc2-conjugated bead using anti-human IgG detection, starting with 500 beads per well but with the instrument programmed to acquire signal from either 5, 10 or 30 beads. As shown in Fig. 3A, the resulting curves were superimposable. A one-way ANOVA test was performed, and no significant differences were observed among the 5, 10, and 30 bead standard curves with P values >0.05.

Another experiment was performed to determine the impact of altering the total number of beads used per well on assay output. This experiment was used to model variation in bead count due to handling, as well as variation in bead count driven by customization of the specific antigen panel used for individual studies. Specifically, it was hypothesized that antibodies may compete for binding to antigen panels with numerous similar HIV env proteins, thus decreasing the signal compared to antigen panels with less potential redundancy, particularly at higher sample dilutions. To mimic the potential effect of the presence of competing bead sets, the assay was performed using different numbers of the same bead across an SIVIG standard curve. Because the same antigen will compete perfectly with itself, this test represents the most extreme case of the potential phenomenon of antigenic overlap. SIVIG standard curves were evaluated against beads conjugated with SIVmac239 gp120 with 500, 5000 and 20,000 beads per well, and detected with a commercial anti-rhesus IgG detection reagent (Fig. 3B). These conditions are equivalent to 1, 10, and 40 times the number of beads normally used in the assay for a single antigen type. The curves were superimposable in the linear range when using 500 or 5000 beads, but a noticeable decrease in signal was observed in the linear range of the assay when 20,000 beads were used. This data suggests that in custom antigen panels, the existence of a moderate number of similar custom antigens (~ten variants) per type can be reasonably expected to have a minimal effect on assay results.

Another possible experimental confounder related to the beads used is mis-classification of spectrally adjacent bead sets. The Flex-Map 3D instrument used for this assay classifies bead types based on the fluorescence signal of three dyes impregnated in the bead during the production process. In theory, each bead type is confined to a unique 3-dimensional region of signal as defined by each dye signal. However, there is the possibility that the instrument can misclassify ‘adjacent’ bead sets, i.e. those for which the bead of interest shares levels of two of the three fluorophores and is only one unit different in the third. To test for this phenomenon, an experiment was performed in which a single bead region was included in the assay, but data was collected for both that region and a spectrally adjacent region. Events recorded for the adjacent, but omitted bead region were uncommon (Fig. 3C); over 40% of wells had no mis-classified beads and the average misclassification rate per well, defined as the number of events reported for the omitted bead region relative to the number of events assigned in the proper channel, was 2.0%. Experiments designed to quantitatively determine whether mis-assignment impacted assay results found no influence of this level of region mis-assignment (Fig. 3D). In conclusion, it was determined that spectrally adjacent bead regions could be used with minimal effect on assay performance.

### 3.5 Optimization of serum/plasma dilution factor

Testing at a consistent sample dilution is useful for conducting cross-study comparisons economically. To determine the best consistent single dilution to be used, serum samples were obtained from 24 human and 8 rhesus vaccine recipients and titrated to graphically determine assay range and identify an optimal dilution for serum/plasma (data not shown). For both species, R^2^ > 0.8 were typically observed between different dilution factors over a broad range of dilutions for both anti-human IgG/anti-rhesus IgG and FcγR detection reagents. For the majority of features, a 1:1000 dilution exhibited acceptable capacity to distinguish between unique samples, falling within the linear range across measurements and between the lower or upper limits of detection. For features detected by C1q, a higher concentration of antibody was generally necessary for the signal to be within the linear range, and a 1:250 dilution was determined to be optimal. The suitability of these “optimal” dilution factors will clearly depend on the response magnitude present in the samples being tested, but the dilution values described here are a useful starting point for study-specific range-finding experiments, and for enabling comparison across studies using a single, consistent test condition. There may be no single sample dilution that falls within the linear range for every sample and for every antigen evaluated. In cases where this dilution is not optimal due to an unusually high or low response magnitude, or a very uneven response across antigen types, additional sample dilutions can easily be evaluated.

### 3.6 Specificity

**Fig. 4:**
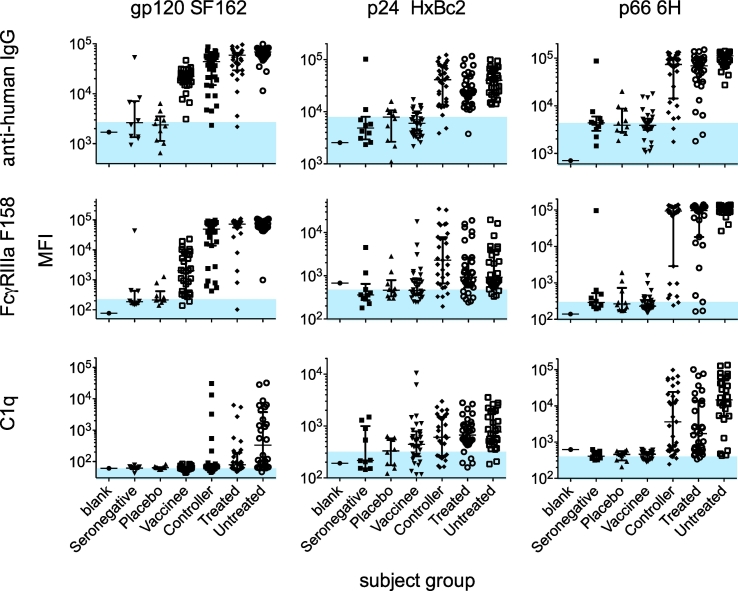
Specificity and range of responses. Signal intensity values for individuals from each subject group are plotted against a three antigen panel (gp120 SF162, p24 HxBc2, and p66 6H) with three detection reagents (anti-human IgG, FcγRIIIA F158 and C1q). Results are a representative subset of 40 antigen-specificities detected with 10 Fc detection reagents. The position of blue shading indicates the median level of signal seen in the HIV-negative (seronegative or placebo) subject groups. Error bars represent the median and interquartile ranges. (For interpretation of the references to colour in this figure legend, the reader is referred to the web version of this article.)

An assay is specific when it can unambiguously detect and uniquely characterize the analyte of interest in the presence of other components, and conversely when it does not detect the analyte of interest in its absence. We have previously demonstrated the ability of the tetrameric FcγR detection reagents used in the Fc Array to show expected profiles in Fc recognition, such as ablated signal when using a NQ mutant Fc and higher signal for IgG1/3 monoclonal antibodies as compared to IgG2/4 with the same Fv specificity (Brown et al., 2017). For qualification of the Fc Array, specificity was defined as whether known positive and negative biological samples could be distinguished. For this task, differentiation between HIV^+^ and HIV^−^ subjects, or vaccine versus placebo recipients is a useful metric of specificity, although true biological positives may not always be available for novel antigens. Therefore, specificity was evaluated using a set of purified IgG from 140 subjects including HIV infected (Controllers, Untreated, Treated), HIV negative (seronegative), and vaccine recipients (placebo and vaccine). These samples were uniformly diluted to a single concentration and evaluated against 40 different antigen types and with ten different Fc detection reagents. To simplify analysis, a subset of three representative antigen-specificities and three detection reagents were presented in detail (Fig. 4). The antigens selected were HIV gp120, p24 and p66 proteins, which show highly different responses and for which there was an expectation that vaccine recipients (immunized with only gp120) should not possess responses against the p24 and p66 antigens. Thus, this set was also selected to cover relevant positive and negative controls across different subject groups. In each plot, a shaded blue region denotes the highest median signal observed for the putative biological negatives (seronegatives or placebos). At the antibody concentration tested, there was generally a two order of magnitude difference in signal magnitude for HIV positive relative to control subjects for all HIV antigen types. Consistent with their presumed lack of antigen exposure, responses against p24, and p66 were not observed among vaccine trial subjects, whether they received placebo or vaccine. A similar experiment was carried out with sham and SIV-vaccinated rhesus macaques to address specificity of the rhesus FcγRs and showed similar results (Brown et al., 2017). Using one set of negative samples to define the baseline and then examining another set for false positives based on the established criteria (≥3SD from technical blank and biological negative controls), overall false positive rates of <10% were observed for both human and rhesus control samples (Table 1).

**Table 1:** Summary of qualification characteristics.

	Experimental design	Observed results
Specificity	HIVIG and Ab samples from 10 HIV− and 90 HIV+ subjects were evaluated to determine specificity. HIVIG and Ab samples from a Rhesus study of 54 HIV env protein vaccinated and 18 negative controls were evaluated to determine specificity.	For both Rhesus and Human specimens, differences of 2 orders of magnitude were often observed between positive control subjects relative to negative control subjects. Titrated controls demonstrated dose-dependent signal. FPR observed = 3.8% for rhesus (for 104 measurements across 16 negative control samples from one study arm, using 2 representative negative control samples to establish baseline). FPR observed = 6.3% for human (for 152 measurements across 6 placebo samples, using 4 representative negative control samples to establish baseline).
Repeatability	HIVIG at a single test concentration was assayed with 21 replicates to evaluate variance. 54 samples from an HIV vaccine study were evaluated in duplicate to determine assay variance.	Average %CVs observed between replicates were ≤10%. The average %CV between replicates of a representative feature was 9.6%, while the average %CV between unique samples was 34.3%.
Intermediate precision	A series of experiments in which the assay was performed by different operators, on different instruments, and/or on different days was conducted to determine assay variance.	Different operators, different instruments, and performance on different days contributed minimally to assay variability (R^2^ ≥ 0.95; %CV ≤16%).
Range/linearity	Sera from multiple human and rhesus subjects were titrated and detected with a representative set of detection reagents.	Signal intensity typically ranged linearly over up to three orders of magnitude (e.g. 200–100,000). Correlations (R^2^) of measurements made at different dilutions were typically >0.8 over a broad range of dilutions (1–2 orders of magnitude), though the dilution range over which good agreement was observed was dependent on detection reagent.
Limit of detection	HIVIG assayed in duplicate across a titration range was utilized to determine pAb detection limits.	Detection limits were calculated per standardized measurement. Ranges of 1–125 ng for LOD were observed for the 5 antigens and 3 detections tabulated.

### 3.7 Precision

**Fig. 5:**
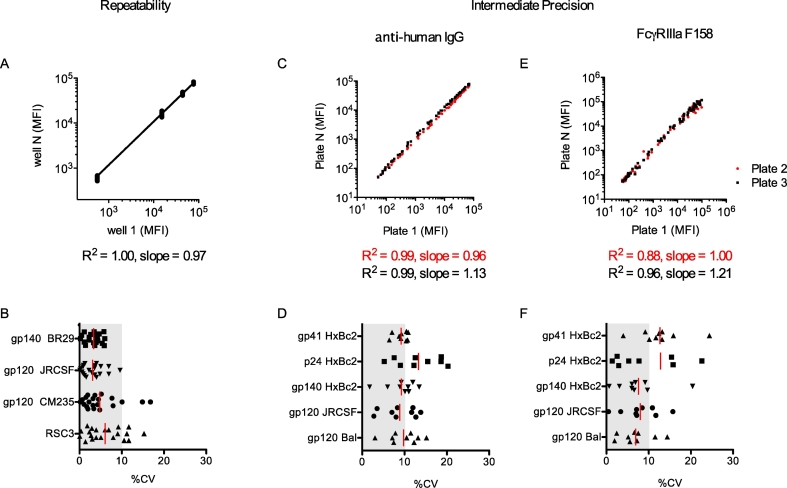
Fc Array repeatability and precision. A. Assay repeatability. Observed MFI values from 21 repeated measurements of HIVIG against a four antigen panel within the same plate. The scatterplot corresponds to the 20 replicates plotted against the values observed in the first well. B. Corresponding coefficients of variation. C—F. Intermediate precision: Observed MFI values from repeated measurements of a HIVIG standard curve against a five antigen panel in experiments conducted by different operators and/or different days with detection by anti-human IgG (C), or FcγRIIIa (E). Scatterplots correspond to second (red) and third (black) replicates plotted against the values observed in the first plate. Corresponding coefficients of variation are shown for anti-human IgG (D) and FcγRIIIa (F) detection reagents. For coefficient of variation plots, bars represent the mean and the gray region denotes %CV ≤ 10%. (For interpretation of the references to colour in this figure legend, the reader is referred to the web version of this article.)

Precision describes the degree of scatter among a series of measurements obtained from multiple tests of the same sample under the prescribed conditions. Precision can be studied at three levels: repeatability (intra-assay variability), intermediate precision (within-laboratory variation), and reproducibility (ability to replicate results between different laboratory sites). As there is currently only one laboratory operating this assay under GCLP conditions, the focus of this section will be on repeatability and intermediate precision. Repeatability was assessed by parallel dilution of 21 replicates of HIVIG to 0.0125 mg/mL and testing for binding to a panel of four antigens (gp120 CM235, gp140 BR29, RSC3, and gp120 JRCSF), as detected by anti-huIgG-PE. These measurements were obtained within a single assay plate (intra-assay variation) and %CVs between wells of 3–8% were observed (Fig. 5A–B). Based on historical data, the variance between positive subjects is typically much greater than the level of variance between these replicates. For example, among 54 samples from vaccinated subjects, average %CV between replicates of a representative feature was 9.6%, while the average %CV between samples for this feature was 34.3% (data not shown).

Intermediate precision was determined both in experiments in which operator and assay day was varied for a set of test samples, as well as by retrospective analysis of actual data collected in the process of analyzing a large study following assay qualification. In this experiment, three sample plates, each containing different samples but a replicated HIVIg standard curve, were assayed for each of eleven detection reagents. Comparison of the three separate standard curves for five different antigen-conjugated bead sets for two representative detection reagents, anti-huIgG-PE (a commercial antibody), and FcγRIIIA (an FcR-streptavidin-PE tetramer produced in-house), showed that plate to plate correlations were excellent (R^2^ values >0.99) for the antibody detection and very good (R^2^ values of 0.88 and 0.96) for the FcγR tetramer (Fig. 5C,E). There was some variability (up to 20%) in the slopes of the best-fit lines, indicating that there may be some systemic bias present from plate to plate. Differences in slope may be due to differences in individual preparations of the detection reagent or slight differences in laser intensity or other acquisition conditions between plates, although further testing showed that instrument variability can be quite low (~5%). Fig. 5D,F shows %CV values for each bead set at each point on the standard curve, giving average CVs of 9.7% for the antibody detection and 13.0% for the FcγR tetramer. Results of these and additional precision experiments in which three individual operators conducted testing on each of three days are summarized in Table 1.

### 3.8 Accuracy

Accuracy is the degree of agreement between the measured value and a value that is either accepted as a conventionally true value, or considered an accepted reference value. Assessing the accuracy of our measurements is made more difficult by a lack of comparative methods for the detection of most antigen–detection reagent pairs used in the assay. In the absence of such data we used results of our EQAPOL (Lynch et al., 2014) external proficiency testing as a proxy for operator and instrument performance, despite the differences in materials and handling. In previous work the array assay was evaluated for its ability to match results from an ELISA assay carried out using the same antigen and detection reagents (Brown et al., 2012). Human FcγRII/III reagents were also tested against a panel of anti-gp120 monoclonal antibodies that were subclass-switched or contained Fc point mutations designed to modify FcγR binding (Lazar et al., 2006; Richards et al., 2008; Shields et al., 2001). Good correlations were again seen between the published affinities determined via ELISA and SPR, and level of binding seen in the array assay (Brown et al., 2017).

### 3.9 Linearity and range

**Fig. 6:**
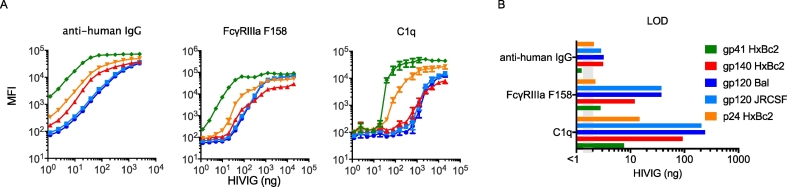
Linearity/range and limit of detection for HIVIG. A. Representative standard HIVIG titration curves for five antigen types and three detection reagents. Each condition was assessed in three independent experiments and error bars represent the mean and standard error. B. Limit of detection (LOD) values for each of these antigen-detection reagent pairs.

Linearity is the ability of an analytical procedure to obtain test results, within a given range, that are directly proportional to the concentration of analyte in the sample. The range of an assay is the interval of concentrations of an analyte in the sample for which it has been demonstrated that the analytical procedure has a suitable level of precision, accuracy, and linearity. For titration curve data this can often be determined by inspection or analysis of curves to determine an appropriate linear range. Retrospective analysis of standard titration curves from a previous study was used to determine the linear range of the assay using HIVIG (pooled positive control) for a representative set of antigens and detection reagents (Fig. 6A, Table 1). Linear range varied widely, from approximately 4 orders of magnitude for the antibody detection reagent, to approximately 2 orders of magnitude for FcγR reagents, to an even narrower range (approximately 1 order of magnitude) for C1q.

### 3.10 Limit of detection

The limit of detection (LOD) of an analytical procedure is the lowest amount of analyte in a sample that can be detected, but not necessarily quantified as an exact value. Per ICH Guidelines (Validation of analytical procedures: text and methodology Q2(R1), 2005), the average plus three standard deviations of technical negative wells was used for detection limits. Retrospective analysis of a previous study was used to determine LOD values for the HIVIG positive control for a representative set of antigens (Fig. 6B, Supplemental Table 1). LOD ranges of 1–126 ng for HIVIG were observed for a number of antigen/detection reagent pairs. These mass quantities are roughly equivalent to 0.0001–0.01 μL of serum. However, because the detection of antibody concentration is not the primary purpose of the assay, results are reported in MFI. The MFI of FcR tetramer reagents results from a combination of the amount and affinity of antigen-specific antibody present and the ability of that antigen-specific antibody to bind to FcRs. In this way, it is meant to approximate the amount of binding an effector cell might be expected to encounter in the context of antibody-opsonized virions or cells expressing the antigen of interest. In general, the linear range for MFI values lies between 200 and 100,000 MFI, and the use of study-specific biological negative controls is recommended as a basis for establishing positivity criteria.

### 3.11 Robustness

The focus of robustness testing is to determine the consistency of the assay under real-life circumstances, in which changes that can occur in standard laboratory conditions, such as differences in the assay operator, the instrument used, and the reagents used, and including variation due to bead conjugation lots, reagent instability, sample instability, among others. Robustness characteristics, some of which may also be considered as measures of intermediate precision, are summarized in Table 2 and some examples are discussed in detail below.

**Table 2:** Summary of robustness characteristics.

Robustness characteristic	Experimental design	Observed results/conclusions
Bead conjugation batches	Multiple independently prepared lots of conjugated beads were compared.	Lot to lot variability between beads was a minor source of assay variation and can be adequately controlled via bridging studies and tracking with Levey-Jennings plots (R^2^ = 0.99; %CV = 5.0%).
Bead set composition	The number of beads per well was varied from 500 to 20,000 in order to capture the expected impact of assaying up to 40 antigen types that are recognized by a single antibody type.	No statistically significant differences in results were observed when use of 10 perfectly redundant antigen bead sets was modeled experimentally. Varying bead set composition was well-tolerated, however considerable changes in composition may impact results and should be investigated if cross study comparisons are desired.
Buffer composition	Buffer compositions representing a ~10% error range (0.9× to 1.1×) were evaluated in comparison to properly diluted assay buffer.	No statistically significant differences in assay results were observed with buffer intentionally diluted over this range (R^2^ > 0.95). A 10% variance is acceptable in the preparation of this material.
Bead counts	The impact of acquiring relatively few beads in each assay well was evaluated.	No statistically significant differences were observed in assay results when ≥5 beads were acquired.
Sample handling/stability	The impact of sample storage and handling was assessed by comparing assay results: A) for a sample of HIVIG which had either been subjected to a freeze-thaw cycle or kept refrigerated. B) for diluted samples that were refrigerated in a master plate for two, four or six weeks or from freshly prepared dilutions.	Specimens may undergo freeze-thaws (R^2^ > 0.95), although care should be taken to reduce the number of these cycles where possible and document their occurrence. Storage of diluted specimens at 4 °C for up to 6 weeks is sub-optimal (R^2^ < 0.8; %CV = 20%). A practice of minimizing storage to <1 month was established.
Pipetting method (robotic vs manual)	An assay performed using robotic automation was compared to a manually performed assay repeated on a separate day.	Robotic liquid handling was determined to be a minor source of assay variation, and both pipetting means are acceptable for use (R^2^ > 0.98).
Bead region mis-assignment	Assays were performed while: A) the instrument was programmed to acquire events in adjacent (but empty) bead regions to determine the percentage of mis-assigned beads. B) beads from adjacent regions but with highly distinct signal magnitudes were included or omitted.	A mis-assignment rate of <3% was observed. Standard curves were unaffected (R^2^ > 0.9) by bead region mis-assignment due to use of beads in a neighboring region.

**Fig. 7:**
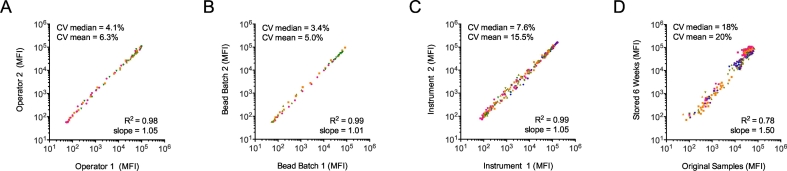
Fc Array robustness. Correlation plots of assay results when operator (A), bead batch (B), FlexMAP instrument (C), or dilution plate age (D) is varied. In each plot, median and mean coefficients of variation are reported at the top left, and correlation coefficients and fit line slopes at the bottom right. Points are colored by antigen, and in each experiment a minimum of four different antigen bead sets were tested.

For well-trained operators there was little difference in assay performance (Fig. 7A). For this experiment operators carried out the assay with the same sample set (a standard curve of HIVIG diluted individually by each operator) on the same day with the same instrument. Curves between operators were comparable for all antigen beads used and individual data points between operators matched with an R^2^ of 0.98 across all measurements (Fig. 7A). In addition, the %CVs between the two operators were below 10% for >75% of points and below 20% for >95% of points (data not shown). A related experiment using a HIVIG standard curve and 13 unique bead sets was also carried out to determine the effect of substituting robotic handling for assay plate setup and addition of reagents following wash steps. This experiment showed that manual and robotic pipetting methods were comparable and both were deemed fit for use for this assay (Table 2). Buffer composition was also shown to not be a major source of variation in a similar fashion, where buffer compositions roughly representing a 10% error range (0.9× to 1.1×) were used in the analysis of HIVIG standard curves. Data obtained using either the 0.9× or 1.1× concentrations of the assay buffer were not statistically distinct from that generated using properly prepared buffer, and gave good agreement (R^2^ > 0.95).

Bead conjugation is a potential source of variation if individual lots of antigen-conjugated beads receive different levels of antigen on their surface. This problem was addressed in three main ways. First, the amount of protein used is in excess of that theoretically needed to fully occupy the surface of the bead. Second, a series of experiments was performed to demonstrate that beads conjugated in different batches, at different times, or by different operators gave comparable results if conjugation and handling of beads was carried out according to the relevant SOPs (data not shown). Finally, new lots of conjugated beads are bridged against the current ‘in use’ set before being used for assays, and the results of all antigen conjugated bead sets tracked over time via Levey-Jennings plots to visualize any changes that occur. A representative bead bridging study was conducted for four antigens (HIV-1 gp120, HIV-1 gp70 V1 V2 scaffold, influenza HA, and an anti-human IgG control bead set) and the results shown in Fig. 7B. For samples whose signal intensities ranged over three orders of magnitude, excellent agreement was observed between independent bead conjugations across all four representative antigen types.

Instrument variability is also a potential factor in results reported in this assay. Accordingly, performance of the assay was tested on multiple Luminex FlexMap 3D instruments of the same model using identical test plates set up and detected simultaneously. Test plates consisted of a twelve point HIVIG standard curve against 13 HIV antigen conjugated beads detected with the anti-human IgG detection reagent. The raw MFI values correlated very strongly (R^2^ = 0.99) with a slope of 0.95, indicating a ~5% systemic bias in signal between instruments (Fig. 7C).

After thawing and aliquoting, samples may be stored in master plates at 4 °C for a period of time prior to assay completion. To test the impact of sample storage on the assay, a set of 15 samples from HIV positive subjects were aliquoted and diluted serially to give three master concentrations with final dilutions of 1:2, 1:20, and 1:200 in assay wash buffer. An assay was performed immediately using this master plate (5 μl of diluted serum +45 μl of bead mix giving final experimental dilutions of 1:20, 1:200, and 1:2000) with a set of three HIV antigen beads, an anti-rhesusIgG detection reagent, and two Human FcγRs. After two and four weeks at 4 °C the same master plate was used as input for an identical assay, using a bead mastermix that had also been stored for six weeks at 4 °C. In this case, data collected when the samples were first thawed was well correlated (R^2^ > 0.9) with measurements made at either the two or four week timepoint. A similar experiment was also performed with SIV-vaccinated and sham rhesus serum samples but was conducted at a six week timepoint, and for stable antigens, differences were minimal between experiments (Fig. 7D). Larger differences were seen for less stable antigens, but this variability was attributed more to stability of the antigens at 4 °C than of the samples themselves. Slopes for best-fit lines varied by antigen, and for samples and antigen specificities giving high signals at the initial timepoint, a systemic bias towards even higher signal at the six week timepoint was noted (best fit line slope of 1.50). Despite reasonable agreement between fresh and stored samples (%CV = 20%), best practice remains to use samples within four weeks of first being thawed to 4 °C.

## 4 Discussion

Here we describe the key parameters and experiments performed to optimize and qualify the Fc Array assay for standardized assessments of the functional capability of HIV/SIV-specific antibodies, although this is by no means an exhaustive list (Table 1). Results from optimization experiments and prior work led to the creation of robust SOPs to comply with GCLP standards. This assay is now being offered as a central service for evaluation of human and non-human primate vaccine trials for HIV/SIV. While the work here focuses on using antibody samples derived from blood, the assay itself has been used to test other sources of polyclonal antibody such as nasal, vaginal, and rectal mucosa samples. However, these other sample types have not undergone the same level of qualification testing as the serum assay, and matrix effects may be present.

One of the goals in the initial design of this assay was to build a system that was both simpler and more robust than current gold standard measures of antibody effector function, typically carried out via a suite of cell-based assays including antibody-dependent cellular phagocytosis (ADCP) (Ackerman et al., 2011), antibody-dependent cellular cytotoxicity (ADCC) (Gomez-Roman et al., 2006), antibody-dependent cellular viral inhibition (ADCVI) (Forthal et al., 1999) and complement-dependent cytotoxicity (CDC) (Hezareh et al., 2001). These assays typically include sources of variation beyond those discussed in this work, particularly in cases in which primary immune cells are used as the effector cell type. Due to additional sources of variation and the general sensitivity of handling primary cells or even cell lines, these assays may also be more sensitive to changes in operator, laboratory site and thus more difficult to qualify or validate in a rigorous analytical fashion. We believe that the ease of use and robustness shown in this work demonstrate a practical advantage of the Fc Array assay over these important biological assay alternatives, even beyond technical advantages such as considerably higher throughput and lower cost.

One limitation of the Fc Array is the difficulty of finding a single set of assay conditions optimized for the diversity of measurements, as each antigen/detection pair behaves differently and may have unique characteristics (e.g. LOD, linearity, range). As such, while general assay performance has been characterized herein, if a more thorough examination of any one feature is desired, a specific analysis of that feature would be recommended to establish positivity thresholds or feature specific ranges, and further optimization of assay conditions (sample dilution in particular) may be appropriate. However, the general conditions outlined here have proven to be useful for the features the assay has been used to capture so far across studies.

Fc Array assay data has already been used in a number of studies for evaluation of both HIV-infected subject cohorts (Ackerman et al., 2016; Lai et al., 2014) and vaccine trials (Barouch et al., 2015; Vaccari et al., 2016; Bradley et al., 2017), and has been shown to be highly predictive of the results of various cell-based functional assays (unpublished data). Data analysis approaches typically involve leveraging approaches from the –omics fields involving supervised and unsupervised machine learning methods to identify similarities in responses and among responders that may be related to differences in outcomes. Ongoing studies will attempt to use these data to better define correlates of protection in HIV and SIV vaccine trials.

The following are the supplementary data related to this article.

Supplemental Table 1 Limits of Detection and Quantitation. Supplemental Table 1
